# Evolution of Engineered ADAR-Based RNA Editing Systems

**DOI:** 10.3390/ijms27041858

**Published:** 2026-02-14

**Authors:** Lidia Borkiewicz

**Affiliations:** Department of Biochemistry and Molecular Biology, Medical University of Lublin, Chodzki 1, 20-093 Lublin, Poland; lidia.borkiewicz@umlub.edu.pl

**Keywords:** RNA editing, ADAR, adenine deamination, RNA-based therapy

## Abstract

RNA editing is a way to diversify, regulate expression, and expand the cell transcriptome. The most common RNA editing is the reversible conversion of adenosine (A) to inosine (I) driven by double-stranded RNA-binding adenosine deaminases (ADARs). As inosine is recognized as guanosine (G) during translation, the RNA editing may result in non-synonymous codon changes. For this reason, ADARs have gained attention as promising enzymes to rewrite mRNA. Many efforts were undertaken to engineer a precise, effective, and controllable ADAR-based system to target certain Adenines on RNA to repair pathological mutations. This review summarizes the advances in ADAR-mediated RNA editing, evolving from systems using antisense oligonucleotides as guide RNA to recruit endogenous or overexpressed ADARs, through more complex setups additionally expressing other RNA-binding proteins, to rational designs harnessing ADARs to convert other nucleotides and amplify the low initial signal. Increasing the specificity and yield of RNA editing, expanding the number of targetable sites, and reducing off-target and bystander activity remain key challenges for these technologies. Improving delivery efficiency across a broad range of cell types, as well as optimizing delivery routes in in vivo studies are also critical to harness them as advantageous tools for both research and therapy.

## 1. RNA Modifications

RNA is a very dynamic molecule predominantly found interacting with other RNA, DNA, proteins, or lipids to preserve its diverse functions in the cell, ranging from housekeeping and regulatory to gene coding [1]. Additionally, through various modifications, RNA is regulated and diversified, shaping the complexity of gene expression. Both coding and non-coding RNAs undergo post-transcriptional processing including 3′ capping and 5′ polyadenylation, as well as chemical modifications of certain nucleosides, e.g., N6-methyladenosine (m6A), N1-methyladenosine (m1A), 5-methylcytidine (m5C), N7-methylguanosine (m7G), and pseudouridine (Ψ), affecting biochemical properties of RNA, its stability, translation, localization, and/or functions, yet not changing the primary transcript [2]. RNA modifications such as insertion, deletion, conversions of cytosine-to-uracil (C-to-U) or adenosine-to-inosine (A-to-I), which can possibly edit codon content, thereby altering the nucleotide sequence, are considered RNA editing [3]. As a consequence, mRNA expression, translation, and protein coding output can be altered [4].

### 1.1. A-to-I RNA Editing

The A-to-I conversion is evolutionarily conserved from bacteria to humans [5,6,7]. In vertebrates, it is catalyzed by double-stranded RNA (dsRNA)-binding adenosine deaminases (ADARs), first identified in the late 1980s in *Xenopus laevis* oocytes as a “dsRNA unwinding activity,” since A-to-I conversion correlated with destabilized dsRNA structures [8,9].

**Figure 1:**
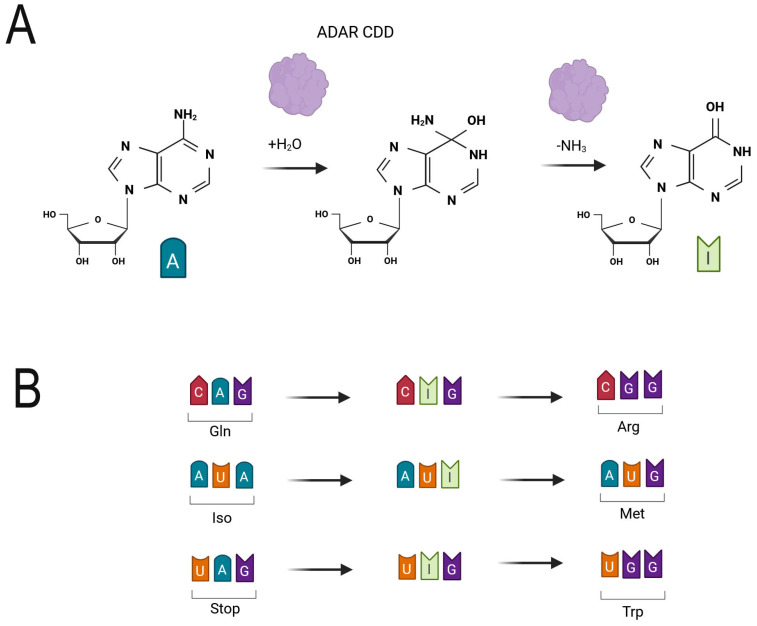
(**A**). ADAR catalyzes hydrolytic deamination of adenosine (A) at the C6 position, converting it to inosine (I), a nucleoside structurally similar to guanosine (G). Consequently, I is recognized by the ribosome as G during translation. (**B**). Examples of ADAR-mediated codon changes in the edited transcript leading to alterations in the amino acid composition of the encoded proteins. C-cytidine, U-uridine.

In humans, two catalytically active ADAR family members, ADAR1 and ADAR2, and one catalytically inactive member, ADAR3, are present [10,11]. ADARs possess a nuclear localization signal (NLS), two-to-three N-terminal dsRNA-binding domains (dsRBDs), and a C-terminal catalytic deaminase domain (CDD) [12]. The dsRBD scans RNA and recruits dsRNA targets for hydrolytic deamination of A at the C6 position by CDD, converting A-to-I (Figure 1), which disfavors interaction with the corresponding U, resulting in unwinding the RNA strand [13,14].

The proper recognition of dsRNA structures mediates initial binding to RNA duplexes and thereby facilitates correct positioning of the CDD [15]. Additionally, 5′ binding loops of ADARs, which reach the edited strand of the dsRNA substrate at phosphates one RNA strand turn 5′ of the edited A, seem crucial for selective preferences of ADARs, as experiments swapping the substantially different ADAR1 and 2, 5′ binding loops revealed [16,17]. For instance, the ADAR2 5′ binding loop favors dsRNA substrates with longer duplexes 5′ of the edited A, while for ADAR1, a minimal 15-nucleotide RNA substrate was established [18]. In addition, ADAR1 is mostly responsible for editing of repetitive sites, while ADAR2 is responsible for non-repetitive coding sites [19].

Also, bases surrounding the edited A are important for both ADARs’ selectivity; in particular, U > A > C > G are preferred bases at the 5′ position relative to the targeted A, while at the 3′ position, G > C ≈ A > U or G > C > U ≈ A [20]. Considering the impact of edited Adenines mismatching based on the ADARs selectivity, an A:C mismatch is favored over A:A, A:G or A:U by ADAR1 and ADAR2 [21].

In addition, dsRNA binding was reported to enhance ADAR’s ability to form homodimers [22,23,24,25]. The crystal structure of ADAR2 revealed that dimer formation facilitates simultaneous contact of a CDD and a dsRBD on the same RNA molecule, and disrupting the interaction by mutagenesis of the dimerization helix inhibits RNA editing in a substrate-specific manner [26].

### 1.2. ADARs Functions

ADAR1, highly expressed in various tissues, exists in two isoforms, ADAR1p110 (110 kDa) and ADAR1p150 (150 kDa), driven by separate promoters [27]. ADAR1p110 is constitutively expressed and primarily involved in nuclear RNA editing, yet represents a non-essential isoform [28,29,30]. In contrast, p150 isoform produced by interferon (IFN)-inducible promoter activity, shuttles between cytoplasm and nucleus, and is indispensable for embryonic development and the suppression of aberrant innate immune activation in mice [27,30]. In fact, ADAR1p150 relocalizes to the nucleus in response to IFN, editing foreign dsRNA targets or endogenous repetitive Alu retroelements commonly found within intronic sequences or 3′ untranslated regions (3′-UTRs). Since the edited RNA is recognized by cellular sensors of dsRNA, such as PKR (protein kinase R) and MDA5 (melanoma differentiation-associated gene 5), as endogenous, ADAR1p150 prevents activation of the IFN pathway and its downstream response—global translation shutdown and cell growth arrest [31]. Also, ADAR1p150 harbors a Zα domain recognizing a left-handed double-stranded Z-RNA resulting from the pairing of inverted Alu repeats, which enhances A-to-I editing, preventing overactivation of ZBP1 (Z binding protein 1) sensor upon autoinflammation [32]. However, upon viral infection, ADAR1p150-mediated editing of foreign dsRNA has been reported to promote viral replication by reducing dsRNA sensing and innate immune activation, as shown in the case of HSV-1, HIV-1, and Influenza A viruses [33,34,35]. On the contrary, extensive editing of hepatitis delta virus (HDV) antigenomic RNA leads to reduced replication [36].

ADAR2 (encoded by ADARB1) in humans is highly expressed in the brain, arteries, lungs, and bladder [37], in two major isoforms: short ADAR2S (or ADAR2a) and the 40-amino-acid longer ADAR2L (or ADAR2b). These are modified further by alternative splicing, revealing ADAR2c and ADAR2d. The specific role of each isoform is not well studied; however, the short isoform exhibits weak activity on dsRNA and in cell lines, such as HEK293 and HeLa, is expressed in higher levels than the longer isoform, suggesting some regulatory function [38]. At the cell level, ADAR2 localizes in nuclei either in nucleoplasm or is sequestered in nucleoli, yet it does not edit nucleolar ribosomal RNA [39,40,41,42]. Studies on mice revealed that nucleolar localization of ADAR2 depends on the presence of rRNA and the binding of ADAR2 dsRBD to RNA duplexes. Yet, in the presence of its dsRNA substrate, ADAR2 dynamically translocates into the nucleus; the transient nucleolar association of ADAR2 may be a mechanism to prevent non-specific activity of ADAR2 in the absence of RNA targets and enables an immediate ADAR2 release to nucleoplasm in response to an increased expression of specific RNA targets [39,40,43].

#### 1.2.1. ADAR RNA Targets

- Neurotransmitter receptors

ADARs activity is essential for neurons’ function as these enzymes target mainly RNAs encoding neurotransmitter receptors, such as ionotropic glutamate AMPA (α-amino-3-hydroxy-5-methyl-4-isoxazolepropionic acid) receptors, kainate receptor subunits GluK2 and GluK1, GABAA receptor subunit α3, and serotonin receptor subtype 2C (5-HT2C) [44,45,46,47]. While in humans mRNA recoding is rather rare, squids and octopuses exhibit widespread ADAR-mediated recoding throughout the brain, located in protein-coding regions [48,49].

One of the best-studied ADAR RNA targets in human neurons is the Q/R site—where a codon for glutamine is converted to that for arginine (Figure 1)—in *GRIA2*, which encodes the glutamate receptor subunit 2 (GLUA2, formerly GluR2), resulting in modification within the pore forming region, important both for assembly with other AMPA receptor subunits as well as for blocking the permeability of GLUA2 to Ca^2+^ ions [44]. Consequently, inefficient Q/R site editing leads to expression of Ca^2+^-permeable AMPA receptors that may cause disorders such as epilepsy in mice and humans [44,50]. An additional target in *GRIA2* is the R/G site (arginine to glycine), edited by both ADAR1 and 2, affecting RNA splicing and such GLUA2 properties as gating, desensitization, and resensitization kinetics of the AMPARs [51,52,53,54].

In kainate receptors, GluK2 Q/R and I/V (isoleucine for valine) sites are edited [45,55,56]. As a consequence of the first site editing, Ca^2+^ permeability and channel conductance are reduced, while conversion of the second modulates channel gating and desensitization kinetics. Interestingly, in GluK2, Q/R editing never reaches 100%, in GluK1, it is performed at a very low level, while in GluK3, it does not undergo editing at all, diversifying the pool of GluK kainate receptors, suggesting a possible mechanism for tuning Ca^2+^ load in neurons [57,58].

ADAR2 also edits voltage-gated channels, in particular potassium channel subunit Kv1.1 and calcium channel subunit Cav1.3 [59,60,61]. Modification of I/V within a region that forms a hairpin structure in Kv1.1 alters channel inactivation dynamics, thereby affecting the channel’s ability to recover to its steady-state conformation, while editing within the IQ domain of Cav1.3 impairs a calmodulin-binding site responsible for inhibitory Ca^2+^ feedback on the channels, thereby increasing cellular Ca^2+^ levels [61]. Interestingly, a spatially diverse pattern of Cav1.3 editing among different neuron populations has been observed, which may reflect ADAR2’s versatile activity across the brain.

Both ADARs target Gabra-3, which codes for the α3 subunit of the GABAA receptor, recoding isoleucine to methionine, therefore changing the architecture of the transmembrane domain α3 subunit, yet the exact effect on the receptor function is to be determined [46,62,63].

Some ADAR RNA substrates undergo editing at a number of sites; for instance, 5-HT2C serotonin receptor mRNA (HTR2C) is edited by ADAR1 and ADAR2 at five specific sites, and the resulting amino acid changes lead to a reduction in the receptor’s G protein-coupling efficiency [47,64] and alter its desensitization and trafficking properties [65,66].

- Other coding and non-coding RNAs

The list of ADAR-targeted RNAs is expanding, including the brain-specific alternative splicing factor NOVA1 [67], CAPS1 (calcium-dependent activator protein for secretion 1), a protein implicated in vesicle exocytosis [68], AZIN1 (antizyme inhibitor 1) [69], and CDK13 (a cyclin-dependent kinase) [70]. Furthermore, ADAR2 drives A-to-I editing of its own mRNA, thereby modulating the alternative splicing patterns [71,72]. In ADAR2, nine independent alternative splicing sites have been reported, where the resulting variants exhibited different activity levels and tissue-specific expression [38,41,73,74,75,76].

ADAR also targets non-coding RNA species such as long primary transcript (pri-miRNA), further cleaved to pre-microRNA, and mature miRNA (e.g., *miR-376* cluster, *miR-455*, *miR-589-3p*), influencing their maturation and target specificity [37,77,78] in brain and immune cells. In addition, several miRNA abnormally edited in various types of cancer, have been reported [37]. For instance ADAR1-edited *miR-200b* promotes cancer cells invasion and migration in head and neck squamous cell carcinoma, kidney renal papillary cell carcinoma, thyroid carcinoma, and uterine corpus endometrial carcinoma [79]; ADAR1-edited *miR-378a-3p* suppresses the malignant phenotype and melanoma metastasis [80], ADAR2-edited miR-376a* inhibits glioblastoma multiforme progression, while its non-edited form enhances tumor invasion and migration in vitro [81].

#### 1.2.2. Role of ADAR-Mediated RNA Editing in Developmental Biology and Disease Pathology

The above examples of ADAR functions highlight the importance of A-to-I RNA editing for evading innate immune responses to endogenous double-stranded RNA and for regulating neuronal maturation and synaptic plasticity. However, RNA-editing also seems to play an important function during the earliest stages of human development [82]. Distinct RNA-editing patterns have been identified in human preimplantation embryos, with many editing events localized within 3′UTRs of maternal mRNAs. These observations indicate a potential role for RNA editing in processes such as maternal mRNA clearance during the maternal-to-zygotic transition, although this mechanism requires further experimental validation.

Conversely, dysregulated RNA editing contributes to a broad spectrum of human diseases. Loss or impairment of ADAR1 function results in abnormal activation of innate immune pathways, driving autoinflammatory disorders such as Aicardi–Goutières syndrome and other autoimmune interferonopathies [83,84]. Reduced editing at the GluA2 Q/R site is linked to amyotrophic lateral sclerosis (ALS) and forms of epilepsy, whereas abnormal editing of the serotonin receptor HTR2C has been associated with neuropsychiatric conditions, including schizophrenia and major depression [85,86,87,88]. Additionally, extensive hyperediting or misediting within non-coding regions can promote tumorigenesis as mentioned above [79,80,81]. Together, these findings underscore the diverse and critical biological roles of RNA editing in both healthy development and human disease.

## 2. ADAR-Based Programmable RNA Mutagenesis

### 2.1. Cross-Disciplinary Aim

As ADARs catalyze site-specific A-to-I editing, they could be engineered to target certain RNAs to repair mutations without the technological and ethical problems related to modifying the genome. What began as a fundamental mechanism governing transcript diversity has inspired a generation of RNA-editing platforms that rely on, redirect, or re-engineer ADAR activity to achieve targeted, reversible changes in living cells, both for research and therapeutic applications.

In research, programmable RNA-editing tools could enable precise manipulation of gene expression, allowing investigators to probe the functional consequences of specific mutations, modulate protein isoforms, correct pathogenic variants transiently, or regulate signaling pathways with high temporal control. In diagnostics, RNA editing could be leveraged to detect foreign dsRNA, while in clinical applications, such technology offers an attractive alternative to conventional gene therapy by enabling correction of disease-causing mutations at the RNA level, restoration of proper protein function, or modulation of aberrant immune and neuronal signaling. Because RNA edits are reversible and alter specific transcripts, ADAR-based therapeutics hold potential for treating a wide range of conditions—including genetic disorders, neurological diseases, and immune dysregulation—while minimizing long-term off-target effects.

**Figure 2:**
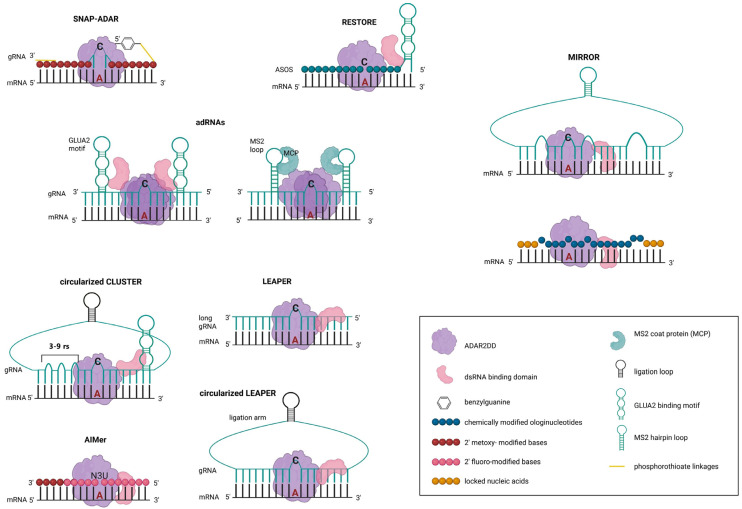
Various guide RNAs are used in ADAR-based RNA editing systems. In SNAP-ADAR, a fusion protein consisting of an ADAR domain (CDD) linked to a SNAP-tag (O6-alkylguanine-DNA-alkyl transferase peptide) is applied with a synthetic antisense oligonucleotide (ASO) bearing a SNAP-reactive moiety (benzylguanine) to recruit the fusion protein to a target mRNA. In RESTORE (recruiting endogenous ADAR to specific transcripts for oligonucleotide-mediated RNA editing), ASOs are fused with ADAR-binding GLUA2 structure to recruit endogenous ADARs. Associated ADAR guide RNAs (adRNAs) are engineered to contain either a GLUA2-binding motif (endogenous ADAR recruitment) or MS2 hairpin loop recognized by MS2 coat protein (MCP) fused with ADARCDD (exogenous ADAR overexpression). LEAPER (leveraging endogenous ADAR for programmable editing of RNA) uses a long RNA (arRNA) complementing a target mRNA to form a large, partially mismatched duplex. CLUSTER uses multiple ADAR-recruiting RNA elements assembled in tandem with a structured RNA motif for ADAR recruitment (GLUA2). Platforms such as CLUSTER, LEAPER, and MIRROR (mimicking inverted repeats to recruit ADARs using engineered oligoribonucleotides), applying a modular guide RNA that mimics ADAR’s natural RNA substrates by adopting defined secondary structures, were circularized to improve stability of gRNA-mRNA binding. In the MIRROR and AIMer approaches, synthetic oligonucleotides mimicking ADAR-recruiting motifs or creating a dsRNA substrate are applied. Mismatching base opposite targeted A is marked—C or N3U (N-3-uridine). In circularized CLUSTER number of repeated structural regions (rs) is marked. Specific modifications are described in the inset.

Therefore, efforts to harness ADAR enzymatic activity for programmable RNA mutagenesis were undertaken and initially aimed to redirect its activity toward specific RNA targets. To that end, ADAR’s dsRNA-binding domain, responsible for target recognition, was truncated from the catalytic domain, which was further engineered with an antisense RNA, complementary to any target sequence, called guide RNA (gRNA) (Figure 2). Alternatively, endogenous ADARs were recruited to new sites by the known ADAR-binding domains fused with gRNA. In parallel, known RNA-binding proteins were fused with the CDD of ADAR to recognize specific RNA sequences fused with gRNA, and were engineered to target RNAs to be repaired.

### 2.2. ADAR–Guide RNA Systems

#### 2.2.1. ADAR Tagging

The first attempts to direct ADAR enzymes for a specific site were based on tagging of catalytic domain of hADAR1 or hADAR2 with a SNAP-tag domain (an engineered O6-alkylguanine-DNA-alkyl transferase peptide) allowing its conjugation with benzylguanine modified gRNA complementary to target sequence with a mismatching C opposite the located in the middle target A, which was common also for the upcoming technologies (Figure 2). This approach was proven effective in *Escherichia coli* for repairing selected point mutations in mRNA encoding enhanced cyan fluorescent protein (UAG in eCFP*STOP66W*, Figure 1) and enhanced green fluorescent protein (UAC and AGC in eGFP *Y65C*, and *S67G*, respectively), with different efficiencies depending on the target site, and the gRNA architecture [89,90]. Since this technology showed a significant selectivity accompanied by no bystander editing on the other Adenines present in the generated dsRNA, SNAP–ADAR provided a foundation for future platforms.

#### 2.2.2. Application of ADAR Recruiting Domains

The different architectures of ADAR-guide RNAs were intensively explored. One of such designs was adRNAs (associated ADAR guide RNAs) composed of a programmable antisense region to the target RNA sequence, with the A:C mismatch located in the middle, flanked by natural or modified ADAR-recruiting domains, engineered from GLUA2 pre-mRNA [80]. Application of adRNAs generated short double-stranded regions that recruited endogenous ADARs, achieving up to 30% RNA-editing efficiency when long (>60 nt) adRNAs were expressed with ADAR2 or its hyperactive isoform *E488Q* in HEK293 cells. Higher yields (up to 50%) were reported for adRNAs with known hairpin structure originating from MS2 bacteriophage, MS2 loop instead of GLUA2 binding domain, recruited by MS2 coat protein (MCP) fused with ADAR1 CDD or ADAR2 CDD *E488Q* (Figure 2). However, in the case of ADAR overexpression, transcriptome-wide off-target effects were found. Nevertheless, the system was packed as a cargo into Adeno-associated virus (AAV) particles and injected into mouse model for Duchenne muscular dystrophy (DMD) to edit *mdx* mRNA bearing an UAA stop codon in exon 23 of the dystrophin gene, as well as in mouse model of ornithine transcarbamylase (OTC) deficiency to correct a G→ A point mutation in the last nucleotide of the fourth exon of the OTC mRNA. The results varied considerably, reaching from 3% (in the DMD model) to 48% editing efficiency in OTC repair when adRNA-MS2 together with ADAR2*E488Q* were applied [91].

#### 2.2.3. Synthetic Antisense Oligonucleotides (ASOs)

On the other hand, RNA-editing platforms engaging endogenous ADARs were found to generate fewer off-target edits in comparison with ADAR-overexpressing systems. RNA editing yielded from 20 to 30%, and no off-target RNA editing was obtained with the administration of densely chemically optimized antisense oligonucleotides (ASOs) to the target site fused with ADAR-recruiting domains from GLUA2, known as RESTORE (recruiting endogenous ADAR to specific transcripts for oligonucleotide-mediated RNA editing) (Figure 2) [92]. To enhance specificity, the oligonucleotide length was optimized (up to 40 nt recognition sequence), and ASOs were modified with 2′-O-methylation, phosphorothioate linkages incorporation, and addition of three locked nucleic acids (LNA) to increase their binding affinity. This strategy was shown effective in *GAPDH* (glyceraldehyde-3-phosphate dehydrogenase) editing in several human cell lines (e.g., HeLa, U2OS, SH-SH5Y, HepG2, AKN1, A549), including primary cells (fibroblasts, HUVEC, HAEC) with variable efficiency (in average 31.5%, reaching up to 62.6% in average after IFNα treatment to activate ADAR1 p150 isoform) and had no off-target RNA editing or other effects on cell endogenous RNA editing processes. Moreover, RESTORE was reported to repair the clinically relevant PiZZ mutation, a single missense mutation *E342K* in α1-antitrypsin encoding gene, *SERPINA1*, leading to the accumulation of the misfolded protein in the endoplasmic reticulum of liver cells, causing α1-antitrypsin deficiency and liver disease, as well as to edit phosphotyrosine 701 in STAT1, signaling factor, required to perform its maximal transcriptional activity, in vitro. These results revealed the therapeutic potential of the approach, especially since the application of chemically modified ASOs as effective drugs was reported previously; however, to use RESTORE in the clinic, further studies on more efficient ADAR recruitment and engagement of other ADARs in vivo will be crucial.

#### 2.2.4. Long Antisense gRNAs

In contrast, ADAR-recruiting gRNAs (arRNAs) designed for the LEAPER (leveraging endogenous ADAR for programmable editing of RNA) system do not contain an ADAR recruitment domain; instead, expressing long (above 60 nucleotides) antisense to the targeted site gRNA alone creates a double-stranded RNA recognized by endogenous ADARs (Figure 2). This can result in efficiency reaching often >50% at the optimized sites, with the strongest and most consistent editing in cells with high endogenous ADAR1 expression, such as HEK293, and moderate editing efficiencies in primary human fibroblasts and T cells [93]. However, while maintaining significant on-target RNA editing, LEAPER was shown to exhibit a certain bystander activity, which can cause some unwanted recoding events in the target, as context-dependent decoding of I as A and, rarely, uracil, was reported [94]. Moreover, since inosine-dependent ribosome stalling during elongation was confirmed in vivo, overediting of mRNA by ADAR enzymes may contribute to this event or even lead to ribosome collisions and trigger a ribotoxic stress response (RSR) pathway. To overcome this obstacle, in the upgraded version LEAPER 2.0, covalently closed circular arRNAs, termed circ-arRNAs, were applied, reaching an average ~3.1-fold higher editing efficiency than their linear counterparts when expressed in cells or delivered as in vitro-transcribed circular RNA molecules [95]. Moreover, while designing circ-arRNAs, pairings of uridines with off-target adenosines were excluded, and off-target editing was almost completely eliminated.

Following the discovery that the local RNA duplex architecture, rather than just the gRNA sequence, is crucial for efficient ADAR recruitment, a CLUSTERed guide RNAs (cgRNAs) were developed (Figure 2). The cgRNAs were composed of multiple (3–9) short (15 nucleotides) ADAR-recruiting regions that can bind adjacent or nearby sequences on the same target RNA, a 20-nucleotide specificity domain complementary to the target sequence, and GLUA2-derived ADAR-recruiting domain [96]. A great advantage of this technology was achieving a significant (up to 45% in HeLa cells, depending on the specific site) RNA editing efficiency while maintaining no or minimal bystander editing, as well as expanding the target repertoire in comparison with single gRNAs. Application of CLUSTER gRNAs in vivo with the dual-luciferase reporter plasmid in the mouse liver resulted in 10% on-target editing efficiency.

#### 2.2.5. Circularized gRNAs

Additionally, efforts to enhance gRNA resistance against cellular exonucleases by either increasing the length to 200 bp, or coupling it with a human U6 small nuclear RNA (snRNA) promoter to drive nuclear expression of a single gRNA, were proven previously to enhance stability of small interfering RNA (siRNA) [97]; finally, engineering circularized adRNA (cadRNA) versions significantly improved efficiency of RNA editing, reaching in the case of the *RAB7A* transcript, encoding a member of RAS oncogene family, up to 1.5-, 2.0-, 3.5-fold change, over the long antisense guide RNAs, accompanied by reduction in bystander editing [98]. Additionally, the significant yield of RNA editing was maintained 48 h and 96 h after transfection via these, in contrast to weakly detectable RNA editing after transfection via linear guide RNAs by 96 h, indicating a good persistence of cadRNAs. As this approach was successful for efficient A editing in 3′ UTRs and coding sequences (CDS) of different mRNAs in vitro, attempts to use it in vivo to repair a nonsense mutation UAG-to-UGG in the *IDUA* mRNA encoding α-ʟ-iduronidase associated with a mucopolysaccharidosis type I-Hurler syndrome were undertaken. Delivery of cadRNA targeting UAG using adeno-associated viruses resulted in approximately 12% correction of the premature stop codon in mice.

A key innovation to suppress the bystander editing, which is a severe limiting factor for many of the developed systems, without losing the highly precise and efficient editing, was the implementation of non-Watson–Crick pairings (wobble base pairings) with a circularized format of the CLUSTER approach [99]. The presence of wobble base pairs in proximity of edited A modulate RNA editing in an orientation-dependent manner and since the bystander editing is driven primarily on such triplets as 5′-UAN triplets (N = A, U, G or C), 5′-AAG and 5′-CAG, introducing G opposite 5′-U or U opposite 3′-G in gRNA will result in G•U wobble pair and reduce bystander editing while maintaining or increasing on-target editing. The destabilization of the duplex and relaxation of local helical structure by the presence of wobble pairs may facilitate base-flipping of the target adenosine into the ADAR catalytic site, or generate a local structure that ADAR recognizes as a target site, resulting in enhanced on-target editing.

Further, circularization of the clustered gRNAs was shown to improve stability and yield of RNA editing, also with respect to other systems such as LEAPER or Cas13-ADAR and λN-ADAR system (described below).

Application of wobble-enhanced circular CLUSTER gRNAs to repair a mutation in the methyl-CpG binding protein 2-Mecp2 mRNA associated with a severe and progressive neurological disorder, Rett syndrome, resulted in up to 87% on-target A-to-I editing with minimal bystander activity in HeLa cells, and up to ~19% editing efficiency across selected brain regions in mice, accompanied by functional restoration of MeCP2 protein and excellent bystander editing control, when delivered with AAVs, highlighting a hopeful step toward therapeutic applications. To that end, further advances—such as improved architecture of guide RNA and its stability, optimized AAV capsid engineering and delivery routes in vivo—will be essential.

Efforts to enhance ADAR efficiency by chemical modifications of the short, chemically modified oligonucleotides (AIMers), which bind to the targeted A surrounding nucleotides, create dsRNA and induce RNA editing by ADAR (Figure 2), revealed that mismatching C opposite the A might not be the best choice [100,101]. As shown in mice, the dense distribution of sugar and backbone modifications, together with incorporating N-3-uridine (N3U, also referred to as isouridine) in oligonucleotides opposite the targeted A, improves RNA editing efficiency by mimicking the RNA–enzyme interactions present in the hyperactive ADAR1/ADAR2 mutants.

Recently, an alternative for short gRNAs applied in the RESTORE approach, or a long, biologically generated gRNA, as seen in the LEAPER and CLUSTER, that engages RNA structural motifs not fully complementary to the targeted site for ADAR recruitment, containing RNA bulges and loops, to enable rational gRNA design, called MIRROR (mimicking inverted repeats to recruit ADARs using engineered oligoribonucleotides) has been reported (Figure 2) [102].

In this technology, gRNAs were modified by the addition of the inverted Alu repeats surrounding the editing site, and binding regions were incorporated at the 5′ and/or 3′ ends of gRNAs, improving target binding. Also, gRNA length was adjusted to fine-tune editing of certain target bases, yet keeping editing sites at the 5′ region of the target and the A target opposite the 3′ region of the gRNA, as this architecture resulted in the highest RNA editing efficiency in mouse cells. Interestingly, the design is applicable to both short chemically modified gRNAs as well as to long biologically generated gRNAs. MIRROR was proven twice more effective in repairing a point mutation in the *SERPINA1* mRNA gene than short chemically modified gRNAs fully complementary to the target sequence in mouse hepatocytes. In comparison of targeting selected three sites (AAG in *TPT1*, translationally controlled tumor protein; CAU in *SRS1*, Silver–Russell syndrome-1; and UAC in *RAB7A*) in five cell types (HEK293T, A549, HeLa, SH-SY5Y, and U2OS), MIRROR gRNAs performed higher efficiency than LEAPER.v2, CLUSTER and circular CLUSTER designs in most cases, showing their applicability in a wide range of cells. Moreover, to simplify the workflow, the authors developed a program, which, after submitting a target editing site and surrounding sequences, automatically identifies the Alu substrates with matching motifs, and creates 1000–2000 recommended gRNAs for high-throughput or top 5-20 gRNAs for low-throughput screening.

### 2.3. ADAR’s Recruitment by Proteins

As mentioned above, the ADAR catalytic domain is capable of deaminating target adenosines without any protein cofactors (in vitro) [103]. To enhance the substrate specificity and strength of the dsRNA binding, several other approaches engaging various known RNA-binding proteins to recruit the ADAR catalytic domain to the target site have been developed.

#### 2.3.1. RNA Hairpin-Loop-Binding Proteins

**Figure 3:**
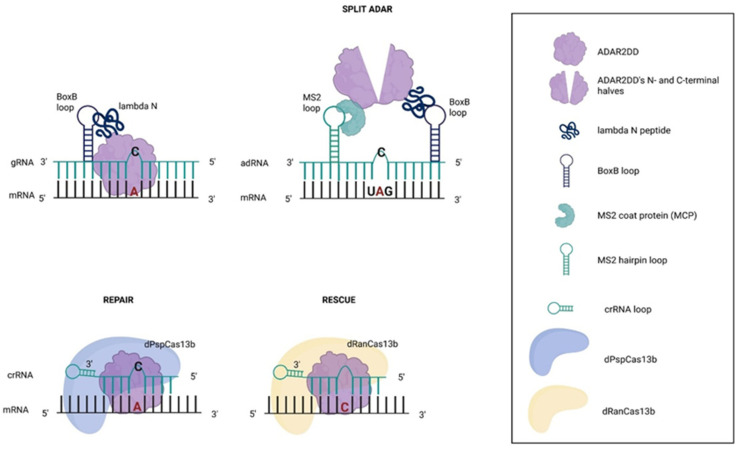
ADAR’s recruitment by RNA-binding exogenous proteins. An engineered guide RNA complementary to the target RNA containing Box B hairpin, which is specifically bound by the bacteriophage λN peptide fused with ADAR catalytic deaminase domains (CDD), enabling tethering of the enzyme to the targeted A. In SPLIT ADAR, the ADAR CDD is split into two inactive halves, each fused with separate RNA-binding proteins: λN or MCP that recognize adjacent sites—the Box B loop and MS2 loop, respectively—on the guide RNA. When both halves are co-recruited to the RNA, the deaminase domain reassembles into an active enzyme, enabling highly specific, proximity-dependent A-to-I editing. REPAIR (RNA editing for programmable A-to-I replacement) and RESCUE (RNA editing for specific C-to-U exchange) use a catalytically inactive CRISPR endonuclease dPspCas13b and dRanCas13b, respectively, fused to ADAR2 CDD. Next, CRISPR RNA (crRNA) guides dCas13-ADAR2 CDD to a target transcript through base pairing, positioning ADAR for precise A-to-I or C-to-U conversion at a defined nucleotide.

The application of specific RNA binding proteins or structure motifs in ADAR-based RNA editing platforms described above as one of the variations in adRNAs was reported also for ADAR2 DD fused to the small bacteriophage lambda N protein (λ N), which specifically binds to the Box B RNA hairpin conjugated with gRNA complementary to the target sequence containing A:G mismatch (Figure 3) [104]. This approach was used to restore the function of mutated cystic fibrosis transmembrane conductance regulator (CFTR) mRNA in vitro and in vivo, in *X. leavis* oocytes reaching 15–20% editing efficiency. Moreover, increasing the number (to two) of Box B hairpins within gRNA, and (to four) λ N fused with ADAR2 CDD, as well as introducing *E488Q* mutation into CDD, further increased editing efficiency in HEK293T [105]. Although Adenines in a variety of different neighboring contexts were edited efficiently, the selected non-target Adenines were also modified with a yield ranging from none to 80%, depending on the target. A reduction in the off-targets was observed when the amount of gRNA used was decreased.

Attempting to have better specificity, ADAR2 CDD was split, and each half was fused to a selective RNA-binding protein, one linked with the MCP and the other to the λN peptide. The complementary gRNA included two flanking loops, MS2 and Box B, thereby complementing ADAR (Figure 3) [106]. This strategy resulted in high transcriptome-wide specificity and a 1000–1300-fold reduction in off-target events compared to full ADAR2 CDD *E488Q*, yet it was accompanied by a 30–50% reduction in editing efficiency.

#### 2.3.2. Cas Endonucleases and Targeting PTM Sites

Genome and transcriptome engineering have undergone a profound transformation with the emergence of CRISPR (clustered regularly interspaced short palindromic repeats) systems, and CRISPR-associated (Cas) proteins were rapidly repurposed as programmable nucleases capable of targeting nucleic acids with unprecedented precision [107,108,109].

Following the success of CRISPR/Cas9 systems for targeted DNA editing, site-directed RNA editing using Cas13 endonucleases, which specifically recognize RNA, was established [110,111]. In parallel, the use of catalytically inactive (“dead”) CRISPR endonucleases to recruit other proteins to DNA or RNA targets was explored [112,113]. For instance, in RNA editing for programmable A-to-I replacement (REPAIR), a Cas13b non-active mutant from *Prevotella* sp. *P5-125* (dPspCas13b) was fused with ADAR2 CDD hyperactive mutant *E488Q* and recruited by a single crRNA (CRISPR-related RNA), forming a hybridized RNA substrate for ADAR2 (Figure 3) [113]. REPAIR was effective in editing 34 disease-related G-to-A mutations with 28% efficiency in targeting A in HEK293FT cells. Efforts to pack the system into AAV particles in order to reduce the size (4773 bp) of dCas13b–ADAR CDD by C-terminal truncations of the endonuclease were successful, yet the first REPAIR version exhibited significant off-target RNA editing. Interestingly, when compared to the Box B-λ N-ADAR system and gRNA-ADAR2 [105,114] alternatives, REPAIR showed the highest efficiency, 89%, while other systems reached 54% and 34.5%. Yet, concurrently, it has the highest number of off-target edits (2111), compared to 1800 of such events for Box B-λ N-CDD ADAR, and 60 for gRNA-ADAR, as shown by transcriptome-wide analysis in HEK293FT cells. As control experiments without RNA-targeting components had shown overlapping off-target events for all three systems, indicating an ADAR CDD non-specific activity, further upgrade of the REPAIR system was focused on destabilizing the ADAR non-specific binding with RNA. Random mutagenesis experiments found the most specific version ADAR2 CDD *E488Q*/*T375G*, exhibiting the highest percent editing of the other mutants with the lowest numbers of transcriptome-wide off-targets, which was applied in the upgraded REPAIR version 2. Remarkably, the lower dosage of dCas13b-ADAR2 *E488Q*/*T375G* (10 ng) had fewer off-targets than the higher dosage (150 ng), suggesting that ADAR load to the cell needs to be carefully administered.

REPAIRv2 was further evolved in vitro to expand the number of substrates for RNA editing by harnessing ADAR to also exhibit a cytidine deaminase activity, resulting in C:U conversion. The idea was based on the fact that human ADAR CDD and *E. coli* cytidine deaminase share structural homology in catalytic core, and available programmable C:U RNA editing systems exhibited limited specificity, off-targets, and substrate restriction to single-stranded RNA. To overcome that, selected residues of ADAR2 CDD contacting the RNA substrate were subjected to rational mutagenesis on an ADAR2 CDD fused to the catalytically inactive Cas13b ortholog from *Riemerella anatipestifer* (dRanCas13b) (Figure 3) [115]. The system reported as RNA editing for specific C-to-U exchange (RESCUE) was shown to target both A and C in different sequence contexts, repairing selected disease-relevant mutations in HEK293FT cells. In addition, RESCUE activated the STAT and Wnt/β-catenin signaling pathways by modulating codons encoding key phosphorylation sites in the β-catenin (CTNNB1) transcript. Under normal conditions, phosphorylation of these residues targets β-catenin for ubiquitination and proteasomal degradation; however, RESCUE-mediated editing prevented phosphorylation, resulting in stabilization of β-catenin and a stimulatory effect on the growth of HEK293FT and HUVEC cells. Compared to REPAIR, RESCUE indeed has improved specificity with retained efficiency. The final RESCUE-S version was optimized by screening ADAR2 CDD mutants at residues interacting with the RNA target, leading to selection of the top specificity mutant S375A. Replacement of the serine hydroxyl group with a methyl group reduced hydrogen-bonding and nucleophilic capacity, thereby eliminating non-specific interactions and side reactions and sharpening substrate discrimination without causing major structural disruption. Compared to REPAIR, RESCUE-S has gained a ten-fold reduction in the off-targets. Notably, targeting both A’s and C’s allows conversion of a wider number of codons, encoding residues also critical for other post-translational modifications (PTMs), such as glycosylation, methylation, as well as common catalytic residues, disease mutations, and protective alleles, expanding the application of the system for research and therapy. The in vivo performance of this system remains unknown.

Targeting of PTM sites was also achieved with an improved SNAP-ADAR approach where the gRNA length was optimized (22 nt antisense and 3 nt non-binding loop) and chemically modified by the inclusion of up to four locked nucleic acid (LNA) building blocks, and the introduction of a bivalent linker (Bis-O6-benzylguanine, BisBG) to enforce dimerization of SNAP-ADAR proteins on guide RNA [116]. The upgraded system was successfully used to modulate JAK/STAT signaling, which, when activated by IFNλ and IFNα, results in phosphorylation and acetylation of many members of the JAK/STAT pathway, starting from the IFN receptors down to the transcription factors. Therefore, depending on the targeted member PTM sites, RNA editing resulted in inhibition of interferon-induced JAK/STAT signaling, or conversely, in an enhanced downstream gene expression. This result suggests that by altering PTM sites, and possibly the active sites of enzymes or the interface of protein–protein interactions, the technology can be used for therapeutic purposes requiring transient modulation of protein function, stability, interaction network, and signaling output, reaching far beyond correcting pathogenic mutations.

#### 2.3.3. Recording A→ I Editing

Besides the potential use of RNA editing by ADAR, these molecular tools have been adopted to sense cellular events such as gene expression in response to stimuli or the time of these events.

**Figure 4:**
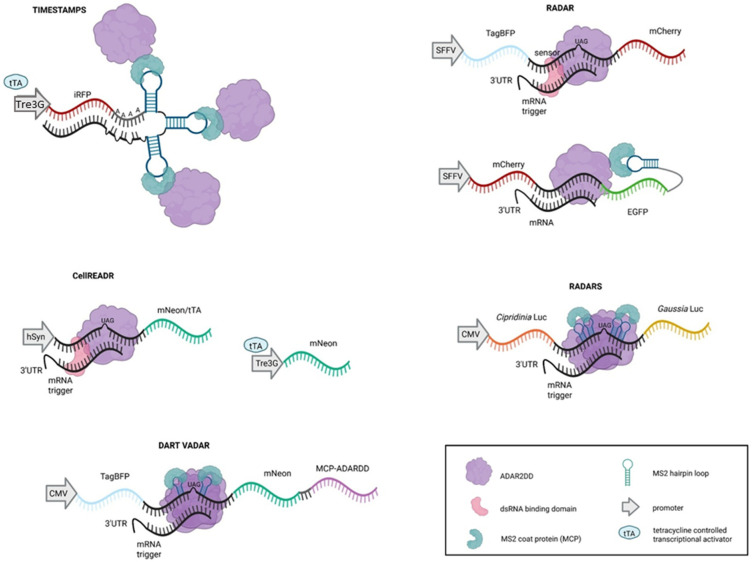
Comparison of mRNA recording and sensing arrays using ADAR-based platforms. The timestamps array consists of A-rich double-stranded regions and an ADAR2-recruiting motif consisting of three MS2 loops recognized by the MCP-ADAR2 CDD complex, expressed in cells. The array expression is triggered by the TetON system (Tre3G promoter) and edited over time, allowing to monitor the age of the mRNA. RADAR (RNA sensors using ADAR), RADARS (reprogrammable ADAR sensors), CellREADR (cell access through RNA sensing by endogenous ADAR), and DART VADAR (detection and amplification of RNA triggers via ADAR) arrays are composed of a sensor complementary to the target mRNA with a strategically located UAG stop codon, flanked by fluorescent protein genes. In the absence of editing, only the first fluorescent protein is translated, while after sensing target mRNA, dsRNA substrate recruits ADAR to rewrite the stop codon, allowing translation of the second fluorescent protein (reporter). In RADAR, RADARS, and DART VADAR, recruitment of ADAR or ADAR2 CDD is enhanced by the introduction of one, two, or four MS2 loops to the array and expression of MCP-ADAR2 CDD fusion protein. In CellREADR and DART VADAR, the low output signal is enhanced either by substituting the fluorescent reporter with the tTA-triggering expression of fluorescent protein or by engaging MCP-ADAR3 CDD *E488Q* downstream of the reporter protein, respectively. The promoters, CMV (cytomegalovirus-derived promoter), SFFV (spleen focus-forming virus), Tre3G (tTA-inducible promoter), and hSyn (human synapsin 1 gene promoter), are marked as gray arrows.

With the trove of information gathered from single-cell transcriptomics, RNA signatures have started to be assigned to various cellular contexts, both physiological and pathological. One of the attempts to measure the age of individual RNA molecules was based on a genetic system recording A→ I editing events accumulating over time, called RNA timestamps [117]. The timestamp array was engineered for adenosine-rich double-stranded regions and ADAR2-recruiting motif consisting of three MS2 RNA loops, targeted by MCP-ADAR2 CDD (Figure 4). As A→ I edits accumulate over time, harvesting cells after different timepoints followed by sequencing allows us to infer time from the moment of stimulation, as the expression of the timestamp RNA is under the Tre3G inducible promoter, triggered by tTA transcriptional activator upon doxycycline. Comparing editing profiles for timestamp RNAs of known ages—specifically the bioinformatic reconstruction of transcriptional dynamics of observed edit distribution in distinct time windows—enables monitoring transcriptional activity in an hour-scale resolution.

#### 2.3.4. Sensing mRNA

In order to detect the expression of particular mRNA in cells, the RNA sensors using ADAR (RADAR) system encodes a sensor sequence complementary to the target RNA of interest (“trigger”) containing a strategically UAG stop codon downstream of a fluorescent protein marker coding sequence, followed by a reporter coding sequence (different fluorescent protein) (Figure 4) [118]. The sensor sequence has the editing-enhancing A:C mismatch within the targeted site. In the presence of the “trigger,” a local double-stranded structure is formed, recruiting endogenous ADAR enzymes, which edit the STOP codon and trigger transcription, resulting in translation of the second reporter. RADAR systems have been used to sense diverse exogenous and endogenous mouse and human transcripts in HEK293T cells, in particular the 3′UTR of the transcripts selected due to the increased ADAR activity on 3′UTRs rather than CDS, likely by the presence of translating ribosomes. Moreover, the expression of RADAR was optimized to reduce the so-called baseline editing observed without “the trigger” by the choice of the optimal constitutive promoter, which was the spleen focus-forming virus (SFFV) promoter. Further optimization was achieved by shortening the sensor, using a split design containing two separated by RNA loops complementary to the same trigger, and sensing CDS’s, which, in some cases, were also effective, enabling sensing of >85% of human and mouse transcripts in vitro. Yet, the highest efficiencies were observed when ADAR1 p150 was overexpressed with RADAR, or when ADAR2 CDD *E488Q*-MCP fusion was used, and an MS2 loop was inserted into the corresponding sensor RNA (Figure 4). However, RADAR sensors against endogenous mRNA showed limited performance; for example, the input sensitivity reached 34–59% of wild-type *GAPDH* expression and ~7% for *DNAJB1*, a member of the HSP40 heat-shock response protein family, suggesting RADAR is functional for only a subset of the transcripts. Nevertheless, its potential for tracking dynamic transcriptional programs across diverse cell types, including plants, deserves further exploration.

Two similar systems, RADARS (reprogrammable ADAR sensors) and CellREADR (cell access through RNA sensing by Endogenous ADAR), have been engineered following the same transcript-triggered ADAR recruitment principle but using slightly different architectures (Figure 4) [119,120]. In the RADARS, an optimized gRNA (ogRNA) containing a UAG stop codon bears five binding sites for the gene of interest, interspersed with four MS2 hairpin loops, which generate a secondary structure that reduces self-folding and enables multivalent binding, serving as a sensor, while *Gaussia Luciferase* or *mNeon* fluorescent protein serves as a reporter [119]. The system is activated in the presence of the mRNA hybridizing to ogRNA, recruiting endogenous ADAR or, more efficiently, overexpressed ADAR1p150, which rewrites the stop codon and allows translation of the reporter gene. Interestingly, introducing MCP-ADAR2 CDD *E488Q/T490A* to the RADARS resulted in a lower on-target editing rate and higher background editing when compared to ADAR1p150 overexpression. The system was tested for the detection of various differentially expressed genes, mostly in HEK293FT cells, and in diverse applications, e.g., multi-input sensing, inducible cell death, in which the *iCaspase* gene was used as a reporter, or triggering liver-derived HepG2 cell-specific apoptosis. Moreover, when Cre recombinase, able to recognize loxP sites in DNA and recombine them, was used as a reporter, after target binding and RNA editing with RADARs, the recombinase specifically targeted the *SERPINA3* gene flanked with loxP in HepG2 cells. These results showed that ogRNA and cargos can be flexibly assembled to achieve cell-type-specific responsive systems using RNA editing.

In CellREADR, the sensor domain comprises around 300 bp, complementary to a specific cellular RNA through sequence-specific base-pairing, and contains one or two ADAR-editable stop codons that act as a translation switch for a downstream reporter gene (sense–edit–switch RNA or sesRNA) [120]. This system allows for the detection of various endogenous mRNAs (e.g., *EIF1A*, *ACTB*, *XIST*, *ARC*) in cells, performing with variable efficiency depending, most probably, on the sesRNA architecture and target mRNA abundance. It is worth noticing that CellREADR is able to detect two different RNAs within the same cell. More importantly, CellREADR was delivered to specific cell types in animal tissues, such as the mouse cerebral cortex, by AAV vector injection, with specific sesRNA targeting mRNA of distinct markers of layers of corticofugal projection neurons and triggered expression of mNeon in neurons expressing the targeted RNA.

Further modification of the system by the use of tTA as a reporter gene, *tTA*, which triggers Tre3G-driven mNeon expression and amplifies the initial signal (second reporter, Figure 4), showed that by targeting the specific GABAergic transcript *VGAT* (vesicular GABA transporter), CellREADR can be applied to monitor neocortical GABAergic neurons in human and rat cortical tissues cultured ex vivo with high specificity (76–94%) and without altering cellular morphology [120].

Although the above circuits using endogenous levels of ADARs were validated in the nervous system, which is known to express high levels of ADAR, their application in other tissues might require enhancing the amount of editing enzymes. This paradigm was studied with the autocatalytic DART VADAR (detection and amplification of RNA triggers via ADAR) sensors, where the RNA sensor sequence complementary to the target RNA sequence contains a UAG stop codon flanked by two MS2 hairpin loops upstream of a reporter coding sequence, which in this case is *mNeon* followed by MCP-ADAR CDD *E488Q* (Figure 4) [121]. Thereby, upon sensing the target RNA, endogenous ADARs induce the expression of MCP-ADAR CDD *E488Q*, which in turn amplifies the initial signal by further editing of the sensing RNA. Such an autocatalytic positive feedback loop could overcome the low background editing by natural ADARs and improve specificity. Indeed, DART VADAR was successfully used to detect a single-base mutation (*Y220H*) in the human P53 tumor suppressor gene associated with the development of breast, lung, and liver cancers, in HEK293FT cells overexpressing P53 *Y220H* in comparison to wild-type cells [122]. Moreover, DART VADAR was shown to discriminate between two closely related lineages of C2C12 murine myoblast cells, able to differentiate to myotubes or osteoblasts, by targeting the 3′UTR of marker genes of these lineages, underlying its potential for diagnosis, e.g., for distinguishing cancer cells from normal cells.

## 3. Discussion and Conclusions

**Figure 5:**
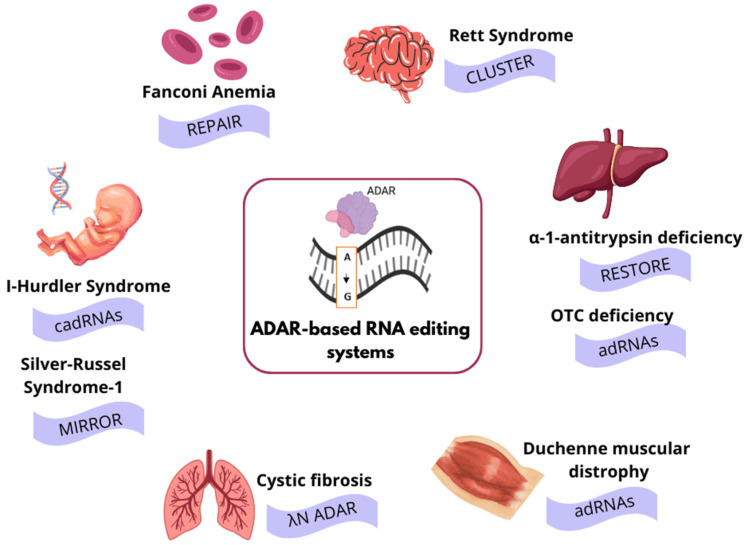
Applications of ADAR-based engineered RNA editing systems in genetic, metabolic, and neurological disease models. A- adenine, G- guanine.

RNA editing technologies offer a therapeutic correction of disease-associated mutations. As RNA editing is inherently transient, edited transcripts undergo natural turnover, thereby enabling reversible modulation of gene function without permanently altering the genomes and minimizing the risk of side mutations. Recently, several ADAR-based RNA editing platforms that rely either on guide RNA (gRNA)-mediated recruitment of endogenous ADARs or on the co-delivery of gRNAs with engineered ADAR variants, including full-length ADARs or ADAR domains fused to auxiliary RNA-binding proteins, have been developed. With the wide range of gRNA architectures, these editing platforms have demonstrated several important achievements as they substantially improve on-target editing efficiency, reaching robust RNA correction in a variety of cell types and animal models. Moreover, the development of engineered ADAR variants with enhanced catalytic activity or improved substrate selectivity has expanded the range of editable sites, including As and Cs, located within suboptimal sequence contexts and residues critical for post-translational modifications. Since several of the engineered systems have been applied across various disease models (Figure 5), the therapeutic potential of these systems is debated.

One major challenge is off-target editing and bystander editing, which can arise from promiscuous ADAR activity on endogenous double-stranded RNA structures or imperfect gRNA-target hybridization. Although rational gRNA design and engineered ADAR variants have reduced unintended edits, comprehensive control over editing specificity has yet to be achieved. Though, due to the transient nature of RNA, even off-targeting effects would have time-limited effects that might be minimal compared to the potential benefits and might not be of importance in some cases, e.g., when triggering cell death.

The second limitation involves the innate immune sensing of dsRNA, as ADAR-recruiting domains in engineered gRNAs can activate cellular sensors of dsRNA and trigger IFN responses. Although chemical modifications and structural optimization can reduce immunogenicity, avoiding unwanted immune activation continues to be a hurdle.

Efficiency also remains variable across target contexts, with editing outcomes influenced by local RNA secondary structure, sequence composition, and cellular ADAR expression levels. In addition, efficient in vivo delivery of gRNAs and ADAR constructs, particularly for tissues with limited transduction efficiency or rapid RNA turnover, is of great interest. Currently, ASOs and AAV-mediated delivery are the most advanced strategies for treating genetic disorders, neurodegenerative diseases, and muscle dystrophies [123]. The targeted distribution may be achieved by engineering the AAV capsid to expose in specific tissues [124]. Nonetheless, safety issues must be addressed, especially considerations on the immune response to AAV vectors, such as precluding the second administration in the same patient, and possible severe adverse events. However, the immunosuppression regimens, use of immune orthogonal AAVs, and capsid engineering to enable lower doses can be advantageous in minimizing the above responses [125].

The approach may be therapeutically useful in cases where transient gene repair is sufficient, for example, when the healthy protein is required only during a specific developmental window, or when short-term restoration triggers durable effects. This is particularly relevant for pediatric disorders, such as Spinal Muscular Atrophy (SMA), caused by *SMN1* mutations critical for motor neuron development [126]. It may also apply to certain cancers, where brief restoration of genes like *TP53* can induce apoptosis or remodel signaling pathways, as shown in lymphoma and acute myeloid leukemia (AML) [127,128]. Finally, current platforms are largely restricted to A-to-I (G) conversions, limiting their utility for broader RNA recoding applications. Continued optimization of gRNA architecture, ADAR engineering, and delivery strategies will be essential to overcome these limitations and fully realize the therapeutic potential of RNA base editing.

In conclusion, the rapidly evolving field of epitranscriptomics is driven by advances in experimental and computational methods for detecting, mapping, and quantifying RNA modifications [129,130]. Recent progress in nanopore-based direct RNA sequencing and mass spectrometry has improved the accuracy and throughput of mapping chemical modifications while reducing sample requirements. Due to the fact that epitranscriptomic marks can regulate RNA metabolism in cancer and other diseases—including responses to chemotherapy—the therapeutic significance of such technologies is increasing [131,132]. Insights into how RNA modifications affect mRNA vaccine stability and efficacy are also critical for RNA-based therapeutics [133]. Growing datasets on modification patterns and their regulators are enabling new diagnostic applications, including biomarker development [134]. Collectively, these collaborative advances underscore the promise of deeper investigation into the analysis and engineering of the epitranscriptome in the years ahead.

## Data Availability

No new data were created or analyzed in this study.
